# Human genome-wide measurement of drug-responsive regulatory activity

**DOI:** 10.1038/s41467-018-07607-x

**Published:** 2018-12-21

**Authors:** Graham D. Johnson, Alejandro Barrera, Ian C. McDowell, Anthony M. D’Ippolito, William H. Majoros, Christopher M. Vockley, Xingyan Wang, Andrew S. Allen, Timothy E. Reddy

**Affiliations:** 1 Center for Genomic and Computational Biology, Duke University, Durham, NC 27708 USA; 2 Department of Biostatistics and Bioinformatics, Duke University Medical Center, Durham, NC 27710 USA; 3 University Program in Genetics and Genomics, Duke University, Durham, NC 27708 USA; 4 Computational Biology and Bioinformatics Program, Duke University, Durham, NC 27708 USA; 5 Present Address: The Broad Institute of MIT and Harvard, 415 Main Street, Cambridge, MA 02142 USA

## Abstract

Environmental stimuli commonly act via changes in gene regulation. Human-genome-scale assays to measure such responses are indirect or require knowledge of the transcription factors (TFs) involved. Here, we present the use of human genome-wide high-throughput reporter assays to measure environmentally-responsive regulatory element activity. We focus on responses to glucocorticoids (GCs), an important class of pharmaceuticals and a paradigmatic genomic response model. We assay GC-responsive regulatory activity across >10^8^ unique DNA fragments, covering the human genome at >50×. Those assays directly detected thousands of GC-responsive regulatory elements genome-wide. We then validate those findings with measurements of transcription factor occupancy, histone modifications, chromatin accessibility, and gene expression. We also detect allele-specific environmental responses. Notably, the assays did not require knowledge of GC response mechanisms. Thus, this technology can be used to agnostically quantify genomic responses for which the underlying mechanism remains unknown.

## Abstract (web summary)

Quantification of genomic responses to environmental stimuli by current genome-scale assays is limited to indirect measurements or requires knowledge of the transcription factors involved. Here, the authors use genome-wide high-throughput reporter assays to agnostically map enhancer activity in response to glucocorticoid treatment across the human genome.

## Introduction

The human genome encodes transcriptional responses to environmental signals. Assaying those responses remains challenging unless the response pathway is well known and reagents have been developed to probe those mechanisms. For example, chromatin immunoprecipitation (ChIP) relies on antibodies against a known transcription factor (TF) to identify environmentally responsive genomic sites. Alternatively, DNase-sequencing (DNase-seq) and ATAC-seq to measure changes in chromatin accessibility, or ChIP-seq for activation-associated histone modifications can also reveal environmentally responsive genomic loci. However, those measurements do not distinguish primary from secondary responses^1–4^. In either case, changes in TF occupancy or in chromatin state often correspond weakly with changes in regulatory element activity^5–7^. In contrast, reporter gene expression assays directly measure *cis*-regulatory activity of DNA fragments without requiring knowledge of the response mechanisms. Despite rapid advances in high-throughput reporter assays, their scale has been limited to targeted regions of the human genome^7–9^ or studies of steady-state cells^10^.

In this study, we present the use of high-throughput reporter assays to measure environmentally responsive regulatory element activity throughout the human genome. To measure regulatory responses genome-wide, we develop a self-transcribing active regulatory region-seq (STARR-seq) plasmid reporter library containing >10^8^ unique DNA fragments from the GM12878 genome. We use this library to quantify regulatory activity in response to glucocorticoids (GCs), an important class of pharmaceuticals and a paradigmatic genomic response model. Our high-throughput reporter approach detect thousands of GC-responsive regulatory elements genome-wide. We integrate these results with complementary genomic datasets collected from the same model system. Changes in regulatory element activity, as measured by reporter assays, correlated with changes in gene expression, histone modifications, and transcription factor occupancy. We also report that stimulus-responsive regulatory activity was only moderately correlated with changes in chromatin accessibility, demonstrating that our approach is orthogonal to existing genome-wide functional genomics assays. Additionally, we were able to identify and validate instances of allele-specific drug-responsive regulatory activity. These results demonstrate that our high-throughput reporter assay approach provides complementarity with widely used genomic assays and the means to quantify en masse aspects of chromatin regulation unavailable to existing technologies. Notably, the results of this study did not require knowledge of GC response mechanisms. Thus, this study provides a demonstration that high-throughput reporter assays can agnostically and quantitatively measure environmentally responsive regulatory element activity across the entire human genome even when an underlying mechanism remains unknown.

## Results

### Whole-genome STARR-seq library

**Fig. 1:**
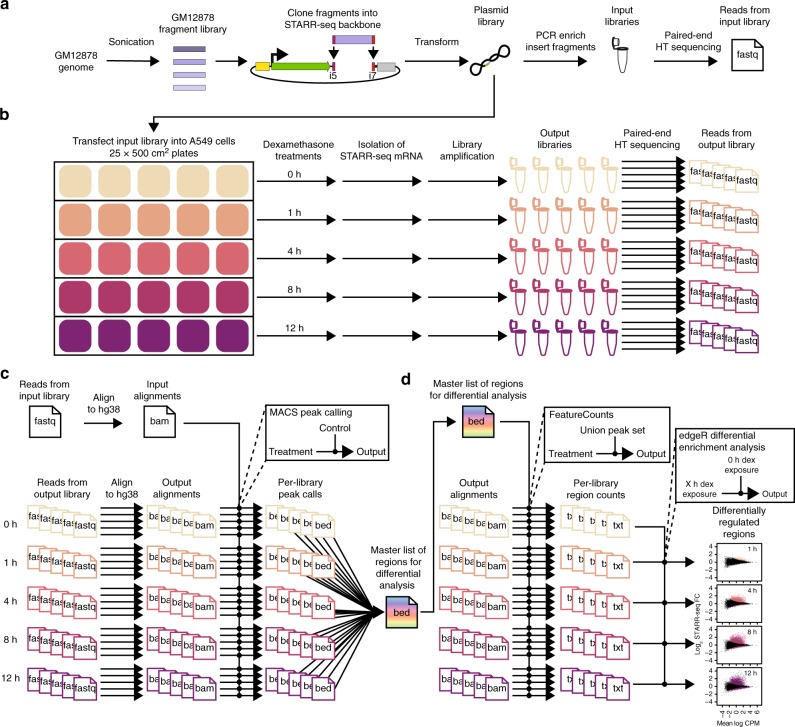
Genome-wide high-throughput reporter library and experimental design. **a** We sheared GM12878 genomic DNA to ~400 bp and cloned into the 3′-UTR of the STARR-seq reporter gene^12^. STARR-seq input DNA-seq libraries were generated by PCR enrichment of the plasmid library (*n* = 12). **b** The whole-genome STARR-seq plasmid library was transfected into 500 cm^2^ plates of A549 cells (*n* = 25). Plates were treated with 100 nM dex or vehicle for a specified amount of time. Total RNA was harvested from five replicate plates for each time point. Following purification, each RNA sample was used to construct STARR-seq output libraries. **c** Following deep sequencing of the input and output libraries, we detected regions of STARR-seq activity with MACS, as is typical for ChIP-seq, to identify regions with significant coverage in each output library relative to the common input library. **d** All called regions from all replicate output libraries were combined to generate a union set of STARR-seq regions. Per-library read counts in regions in the union set were generated with featureCounts. Differential enrichment analysis for each time point relative to 0 h of dex exposure was performed on the union regions set with edgeR prior to downstream analyses

**Fig. 2:**
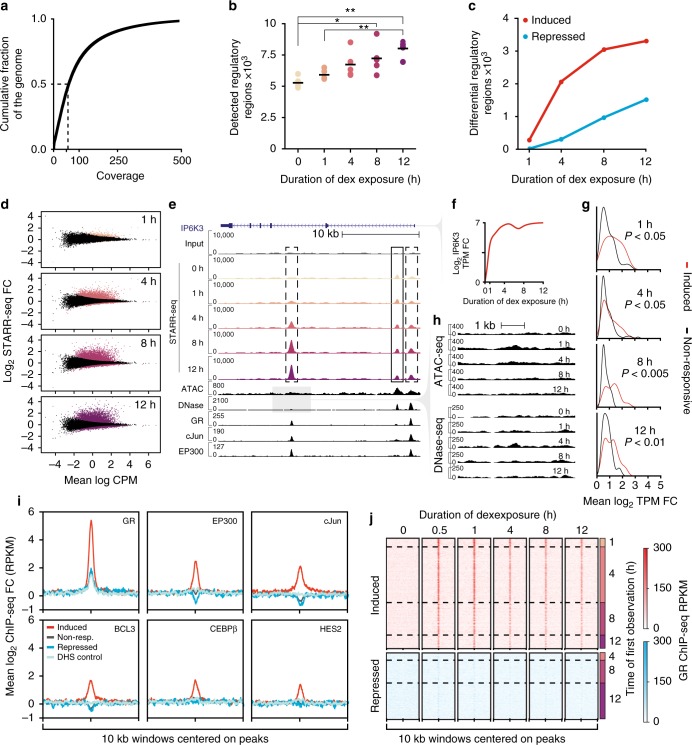
Genome-wide high-throughput reporter assays measure environmentally responsive regulatory element activity. **a** Cumulative distribution of coverage for the whole-genome STARR-seq library. The median per base coverage is 59× (dashed line). **b** The number of regulatory regions identified by STARR-seq in each replicate increased with longer dex exposure (one way ANOVA followed by Tukey’s multiple comparison test; *P < 0.05, **P < 0.01). **c** The number of called DREs (FDR <5%). **d** Distribution of regulatory responses to dex. The fold change in reporter activity for all regulatory regions is plotted as a function of the mean sequencing coverage at that region. Significant responses are colored by time point. **e** Induced (dashed boxes) and steady-state (solid box) regulatory regions detected by whole-genome reporter assays correspond to epigenomic features identified by complimentary genomic assays at the dex-induced IP6K3 locus. STARR-seq input library coverage is displayed in the top track. ATAC-seq, DNase-seq, and ChIP-seq data after 4 h of dex treatment. **f** IP6K3 RNA-seq was measured in transcripts per milion (TPM). **g** GC-responsive gene induction is greater in TADs containing induced DREs compared to TADs containing non-responsive regulatory elements. The distribution of mean fold changes for all differentially induced genes in the same TAD is plotted for all TADs containing an induced DRE or non-responsive regulatory element in DHS. Median fold changes were compared with the Mann–Whitney *U* test. **h** Chromatin accessibility tracks for the 5 kb window centered on the upstream TF-bound induced DRE displayed in **e** (gray box). **i** Aggregate profile plots of the mean fold changes in post-dex ChIP-seq signal across 10 kb windows centered on DHS+ regulatory element midpoints. Induced (*n* = 1646) and repressed (*n* = 746) DREs are shown in red and blue, respectively. Non-responsive regulatory regions (*n* = 6531) are displayed in gray and control regions (*n* = 1646) are shown in pale blue. Control regions are randomly selected 520 bp (median STARR-seq regions length) regions filtered to matched to DHS+ induced DRE accessibility. **j** Heatmaps showing the average GR ChIP-seq signal in 10 kb windows centered on all dynamic DRE midpoints. Rows are grouped according to the time point for which the DRE was first called significant

To quantify the genomic responses to an environmental signal at high resolution, we generated a genome-scale reporter library with ~560 million unique ~400 bp fragments^11^ that covers the genome at 59× (Fig. 1, Fig. 2a, and Supplementary Fig. 1a–c; Supplementary Table 1; see Methods). The library uses the STARR-seq platform in which candidate regulatory elements are cloned into the 3′-untranslated region (UTR) of a reporter gene (Fig. 1a)^12^. From that position, the UTR-embedded elements regulate their own transcription into messenger RNA, and targeted RNA-seq is used to measure activity relative to input control libraries. While other approaches rely on synthesis of candidate regulatory elements, STARR-seq assays use sheared genomic DNA. That difference means that STARR-seq libraries are typically more complex than other high-throughput reporter assay systems, and thus rely on aggregating signal across genomic regions rather than estimating activity of individual DNA fragments. To allow for allele-specific interpretation of our results, we generated the library from the genome of GM12878 cells which has been a major focus of whole-genome sequencing studies^13,14^ and functional genomics studies^15,16^.

### Measuring environmentally induced regulatory responses

We used the whole-genome STARR-seq library to measure genomic responses to an environmental signal. We focused on the response to dexamethasone (dex), a commonly used anti-inflammatory drug that acts via the GC response. Dex binds the ligand-inducible GC receptor (GR), causing it to translocate into the nucleus where it acts as a transcription factor to regulate gene expression^17–19^. To investigate the dynamics of that response, we measured regulatory activity in human A549 cells at 0, 1, 4, 8, and 12 h of treatment (Fig. 1b). We assayed five replicates per time point, with ~3.3 × 10^7^ cells per replicate (Supplementary Table 1). In each replicate, we identified between 4900 and 9200 genomic regions with reporter activity at a false discovery rate (FDR) ≤10% (Fig. 1c, Fig. 2b, and Supplementary Fig. 2a). We then generated an inclusive set of 27,498 regions with evidence of regulatory activity by taking the union of all per-replicate region calls (Supplementary Data 1)^20^. As additional replicate region sets were included in that union, the number of observed regions increased linearly. That suggests we have yet to comprehensively identify all regulatory regions (Supplementary Fig. 2b). However, there was strong concordance between replicates (*R* > 0.91) and a distinction between early (0, 1, and 4 h) and late (8 and 12 h) time points by hierarchical clustering (Supplementary Fig. 2c). Regions exhibiting regulatory activity in the input and output libraries were covered by a median of 57 (SD ± 5) and 36 (SD ± 6) fragments, respectively (Supplementary Fig. 2d, e). Importantly, the replicates provided largely independent assessments of the activity of genomic regions: the majority of fragments in each region were unique to a single replicate, and only about 1% of fragments were assayed in all five replicates of any time point (Supplementary Fig. 2f). Active regions identified by STARR-seq are therefore comprised of output fragments from many replicates demonstrating regulatory activity (Supplementary Fig. 2g).

Prior to identifying dex-induced *cis*-regulatory responses genome-wide, we demonstrated that our genome-scale library recapitulates localized responses. We compared our results to that obtained from two independently constructed bacterial artificial chromosome STARR-seq libraries representing five GC-responsive loci totaling approximately 1 Mb of the human genome (Pearson's correlation coefficient, *r* > 0.83 and 0.91; Supplementary Fig. 3a, b)^7^. To identify dex-induced changes in regulatory activity genome-wide, we evaluated the null hypothesis that dex did not change the activity of each candidate regulatory element at each time point relative to 0 h of dex exposure (Figs. 1d, 2c, d). We rejected that null hypothesis at 5695 genomic regions with an FDR ≤5%, and we refer to those regions as dex-responsive elements (DREs). Dex treatment increased the number of unique fragments corresponding to induced DREs as well as the coverage of those fragments relative to controls (Supplementary Fig. 3c, d). The number of DREs increased with longer treatment, in agreement with RNA-seq data over the same time course (Supplementary Fig. 4a)^21^. Dex responsiveness peaked at 8 h of treatment, even though additional sites had significant responses only later (Supplementary Fig. 4b).

Of the DREs, 3552 (62%) were activated and 2143 (38%) were repressed, similar to the genomic distribution of dex-responsive gene expression^19^. However, unlike for gene expression responses, induced regions had a 1.2- to 1.5-fold greater response to dex than repressed regions (Fig. 2d and Supplementary Fig. 4c). Induced sites were rarely detected as having regulatory activity prior to dex. However, that likely reflected reduced statistical power to detect low pre-dex activity, and regulatory activity was enriched at those sites overall (Mann–Whitney, *P* < 10^−10^; Supplementary Fig. 4d).

### Comparing STARR-seq and orthogonal genomic assays

Dex-responsive reporter activity corresponded with transcriptional responses across the genome (Fig. 2e–g). We compared the mean fold change in expression for all differentially induced genes within topological-associated domains (TADs)^22^ containing dex-responsive or non-responsive active STARR-seq elements. Differentially induced genes in TADs with induced DREs increased expression more than genes in TADs without induced DREs (Fig. 2g). As a negative control, no relationship was observed between induced DREs and gene repression (Supplementary Fig. 5a). We also observed that genes with induced DREs in their promoters exhibited greater changes in gene expression than genes with non-responsive regulatory elements in their promoters (Supplementary Fig. 5b). These results suggest that GC-mediated activation is in part constrained by TAD boundaries^22^ and that dex-responsive increases in reporter activity corresponded to increases in gene expression.

Next, we evaluated whether changes in reporter activity reflect changes in chromatin accessibility at the corresponding genomic locations in A549 cells (Fig. 2e). Overall, 2993 (53%) DREs were in a DNase I-hypersensitive site (DHS) observed at any time point from 0 to 12 h of dex treatment (FDR ≤ 5%). Approximately a quarter of DREs in open chromatin (*n* = 728) overlapped a dex-responsive DHS. The remaining 76% of DREs in open chromatin had a greater dex-dependent change in accessibility than non-responsive regulatory elements in DHS (20% increase vs. 12% increase, respectively; Mann–Whitney, *P* < 2.13 × 10^−64^; Supplementary Fig. 6a). For STARR-seq assays that overlap a DHS, the changes in activity observed correspond to the expected changes in chromatin accessibility.

DREs that do not overlap a DHS are often latent regulatory elements that are active in other environments. Specifically, 69% of non-DHS STARR-seq regions overlap an element predicted to be active by the Epigenomics Roadmap project in a different cellular context (Supplementary Fig. 6b). These results highlight the added value of interpreting whole-genome STARR-seq assay results alongside chromatin accessibility data to distinguish active from latent regulatory elements.

Changes in chromatin accessibility in response to a stimulus are often used as proxies for identifying regulatory regions^5,23,24^. To determine if GC-induced changes in chromatin accessibility corresponded to changes in regulatory activity, we compared differential DNase-seq accessibility and differential reporter activity in DREs. The overlap with differential DHS was more prominent for induced than repressed DREs. Specifically, 19% and 3% of induced and repressed DREs overlapped a differential DHS, respectively. For induced regions, DNase-seq signal increased 31% overall, whereas for repressed regions DNase-seq signal decreased 1% overall (Mann–Whitney, *P* < 10^−100^; Supplementary Fig. 6c). Though changes in chromatin accessibility can nominate DREs, the quantitative changes in accessibility and regulatory element activity do not agree well. Reporter activity in response to dex at induced DREs was moderately correlated with changes in chromatin accessibility (Pearson's correlation coefficient, DNase-seq: 0.13 < *r* < 0.29, ATAC-seq: 0.3 < *r* < 0.43; Supplementary Fig. 6d, e). We also observed that 8% of induced DREs (*n* = 298) were never called DHS, yet were bound by a TF at some point during the time course and were enriched for the transcriptional coactivator histone acetyltransferase EP300 (Fig. 2e, h; Mann–Whitney, *P* < 5.42 × 10^−17^; Supplementary Fig. 6f). These results suggest that a fraction of DREs are bona fide regulatory elements even though they were not called by mapping accessible chromatin.

Regulatory activity is dependent on the binding of TFs at *cis*-regulatory modules^25^. To identify TFs contributing to regulatory element activity in A549 cells, we compared reporter activity to ChIP-seq data from a time-course study of the same dex response^21^. In that study, we performed ChIP-seq for seven TFs (AP-1, BCL3, C/EBPβ, CTCF, EP300, GR, and HES2) and five histone modifications (H3K4me1, H4K3me2, H3K4me3, H3K9me3, and H3K27ac) at 12 time points ranging from 0 to 12 h of 100 nM dex exposures. As with DNase-seq results, approximately 52% of DREs were bound by a TF or enriched for modified histones following treatment. Eighty-nine percent of those overlapping sites were also in DHS. DREs not bound by a TF in our model system likely represent latent regulatory elements that are occupied in other cellular contexts (Supplementary Fig. 6b).

Induced and repressed DREs exhibited contrasting patterns of modified histone enrichment and TF binding. The number of TF ChIP-seq peaks overlapping induced DREs increased with regulatory activity and was greater than that at repressed DREs (Supplementary Fig. 7a, b). Following treatment, deposition of histone modifications associated with active chromatin (H3K427ac and H3K4 methylation) predominated at induced DREs relative to other regulatory elements and accessibility-matched DHS(Supplementary Fig. 7c–g). Induced DREs were strongly associated with increased binding and co-occupancy of the GR and EP300, as well as AP-1, BCL3, C/EBPβ, and HES2 (Fig. 2e, i and Supplementary Fig. 7d). Recruitment of the GR to induced DREs persisted throughout the time course and preceded detection of significant reporter activity at these sites (Fig. 2j). In contrast, TF and modified histone enrichment and co-occupancy was depleted at repressed DREs, and GR binding was comparable to accessibility-matched controls (Fig. 2i and Supplementary Fig. 7c–g). This gives further evidence that DREs identified by episomal whole-genome reporter assays and in accessible chromatin are faithful measurements of genomic regulatory responses.

Unlike ChIP-seq assays, genome-wide reporter assays agnostically query regulatory activity and can therefore detect regulatory elements participating in the GC response that do not directly bind the GR. Such sites may reflect secondary effects due to direct regulation of other TFs by the GR. Throughout the time course we observed a small but increasing percentage (2.1–4.3%) of induced DREs in DHS that lacked GR motifs, GR ChIP-seq peak calls, and for which GR ChIP-seq signal was indistinguishable from genomic controls (Supplementary Fig. 8a). Such DREs were enriched for motifs of known GR co-binding TFs such as the AP-1 family (adj. *P* < 7.43 × 10^−12^) and STAT family (adj. *P* < 9.25 × 10^−3^), and had empirical evidence for binding by the AP-1 component JunB (Fisher’s exact test, *P* < 0.015). Members of both the AP-1 and STAT TF families exhibited GC-mediated increases in expression. Upregulation of these factors may contribute to the GR-independent activation of sequences harboring their corresponding motifs (Supplementary Fig. 8b, c; Supplementary Table 2). These results highlight the potential for whole-genome reporter assays to identify secondary regulatory events in a long-term environmental response.

### Motif enrichment in DREs

**Fig. 3:**
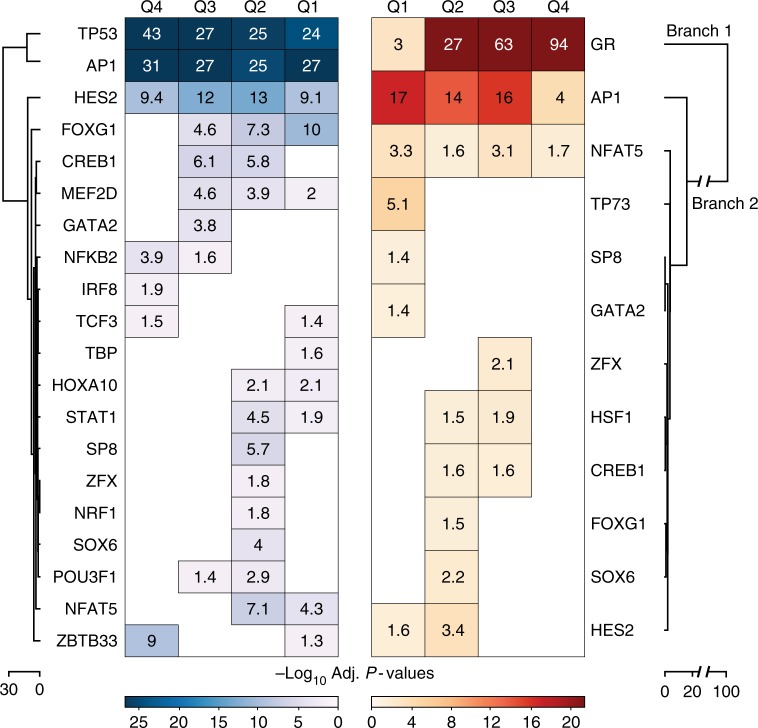
Dex-responsive regulatory elements are enriched for TF-binding motifs. Dynamic DREs identified after 8 h of dex treatment were binned according to their fold change in reporter activity relative to steady state. Enriched motifs were identified for each quartile and then hierarchically clustered. Bonferroni-corrected log_10_ *P* values are displayed for all significant enrichments

We next investigated whether specific TF-binding motifs were enriched in DREs (Fig. 3). Hierarchical clustering on motif enrichment identified groups of motifs enriched at induced or repressed regulatory elements. As expected, induced DREs were associated with GR-binding motifs, especially at the most strongly induced DREs. Those DREs were also enriched for AP-1 motifs. However, AP-1 motifs were most strongly enriched at moderately induced DREs where AP-1 is bound prior to dex treatment (Supplementary Fig. 8d). Elevated AP-1 motif enrichment in the less induced DRE quartiles is contrasted by increased AP-1 binding at more active DREs upon recruitment of the GR (Fig. 2i and Supplementary Fig. 8d, e). These results support previous studies demonstrating synergy between GR and AP-1^7^ and suggest that when GR is bound to strong and multiple consensus motifs it can recruit other TFs to neighboring lower quality motifs. Patterns of motif enrichment at repressed DREs supported the predominance of the GR in regulating the genomic landscape. Repressed DREs were not associated with steroid hormone receptor motifs (Fig. 3), suggesting that the marginal GR occupancy observed at these elements reflects transitory binding at weak motifs or tethered interactions to other TFs^21^. Repressed DREs were more enriched for motifs corresponding to AP-1 and HES factors than induced DREs (Fig. 3), despite AP-1 and HES2 TF depletion at repressed regulatory elements (Fig. 2i). These findings agree with previous studies demonstrating that AP-1 marks enhancers in unstimulated A549 cells whereupon it is redistributed following GC treatment (e.g. ref. ^21^). The enrichment of NF-ϰB motifs at the most repressed DREs is supported by the eviction of this factor after treating with GCs^26^. Dex treatment also repressed regulatory activity from DREs enriched for p53 and interferon factor motif families (Fig. 3). Enrichment of p53 and interferon regulatory factor motifs in steady-state regulatory regions implicates our episomal reporters in eliciting a stress response (Supplementary Fig. 8f)^27–29^. However, the loss in reporter activity at regulatory regions harboring cell-stress motifs following treatment with dex supports the known role of the GR in attenuating the innate immune response^30^.

### Drug-responsive allele-specific regulatory activity

**Fig. 4:**
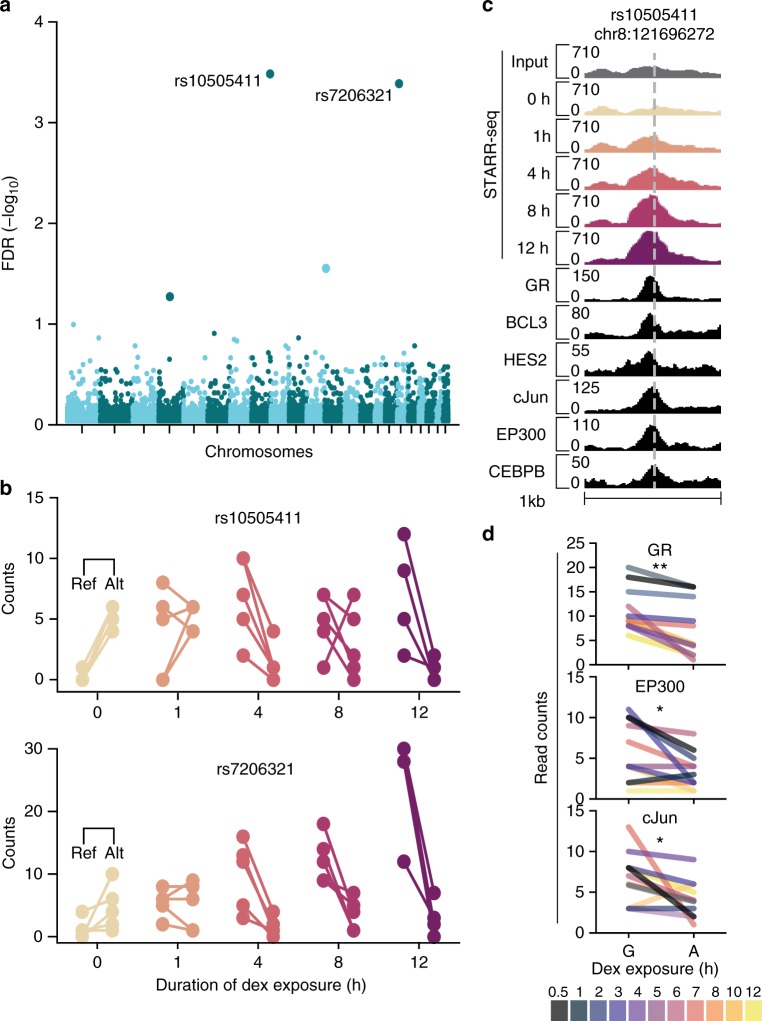
Genome reporter assays detect drug-responsive allele-specific regulatory activity. **a** Manhattan plot of genome-wide significance values for the 10,669 heterozygous SNPs overlapping a regulatory element identified in this study. **b** Replicate read counts at rs10505411 and rs7206321 alleles. **c** Rs10505411 (dashed line) is associated with active epigenomic features in A549 cells. STARR-seq was measured in RPKM and ChIP-seq was measured in input-subtracted RPKM. **d** TF binding at the rs10505411 reference allele is greater than that at the alternate allele following dex treatment. Total pre-dex read counts for each factor were two or less. Wilcoxon's signed-rank test, *P < 0.05, **P < 0.001

By constructing whole-genome STARR-seq assays from the deeply sequenced genome of GM12878 cells, we have the opportunity for allele-specific analyses to detect genetic effects on regulatory element activity. In total, we tested for allele-specific dex-responsive reporter activity at 10,669 heterozygous single-nucleotide polymorphisms (SNPs) found within the genomic regions active in our assay. Two common SNPs, rs7206321 and rs10505411 (minor allele frequency >25%), had statistically significant evidence for allele-specific drug response (Fig. 4a). Prior to treatment, the alternate alleles of both SNPs were more active in our assay. After treating with dex, the trend reversed such that reporter activity at the reference alleles exceeded that at the alternate alleles (FDR <5.0 × 10^−4^; Fig. 4b). Both SNPs were in induced DREs and proximal to a GRE. However, only rs10505411 was associated with significant endogenous epigenomic activity (Fig. 4c). As independent confirmation of the allele-specific dex response in regulatory activity observed at rs10505411, we looked for allele-specific TF binding at the corresponding genomic locus in A549 cells, which are also polymorphic for rs10505411. TF binding at the reference allele significantly exceeded that at the alternate allele for several TFs (GR, AP-1, EP300; Wilcoxon's signed-rank test, *P* < 0.05; Fig. 4d)^21^. Increased TF binding at the reference allele may result from the presence of the variant at the alternate allele converting a GATA3 motif (*P* < 8.37 × 10^−5^) to a FOXG1 motif (*P* < 1.45 × 10^−4^; Supplementary Fig. 8g). Several GATA and FOX factors are expressed in A549 cells. This proof of concept demonstrates that high-throughput reporter assays can identify allele-specific genomic regulatory responses. We anticipate the number of allelic responses that can be detected would increase with inclusion of additional individuals and deeper sequencing in subsequent studies.

In summary, we used high-throughput reporter assays to agnostically and quantitatively measure drug regulatory responses across the entire human genome. Our approach is complementary to other widely used genomic assays, and enables new ways to quantify gene regulatory responses to environmental stimuli. Further, by focusing our analyses on drug-dependent effects, we limit the potential for false positives due to technical biases in high-throughput reporter assays. We demonstrated that whole-genome STARR-seq can detect combinatorial interactions between TFs and identify allele-specific changes in regulatory behavior in response to stimulus. These results demonstrate that whole-genome reporter assays are an effective and new method for studying genomic responses to environmental conditions, particularly when the underlying mechanisms of that response are not well known.

## Methods

### Whole-genome STARR-seq input library construction

Total genomic DNA was extracted from lymphoblastoid cells (GM12878) with the Qiagen DNeasy Blood and Tissue Kit and sheared by sonication on the Covaris S2 system (Intensity: 3; Duty Cycle: 5%, Cycles/Burst: 200; Time: 80 s; volume level: 12). We then performed two-sided size selection with Corning solid-phase reverse immobilization (SPRI) beads. We used 0.4× volume of SPRI beads to remove fragments larger than ~450 bp, followed by a 0.25× volume of SPRI beads to select fragments >350 bp. In 60 parallel reactions, 5 µg of size-selected DNA fragments was end-repaired, A-tailed, and ligated to annealed Illumina TruSeq Universal Adapters using the NEBNext DNA Library Prep Master Mix Kit^12^. Adapted libraries were purified using SPRI beads (1.8× volume to retain most fragments) and subjected to 10 cycles of PCR enrichment using the NEB Q5 PCR Kit with GC buffer and STARR-seq adaptor primers^12^ under the following conditions: 98 °C for 30 s, followed by 10 cycles of 98 °C for 10 s, 63 °C for 30 s, 72 °C for 30 s, with a final extension at 72 °C for 5 min. Amplified inserts were purified using SPRI beads (0.9× to remove primer dimers) and pooled.

Purified PCR products were cloned directly into linearized STARR-seq screening vector as described previously^7,12^ using the NEBuilder HiFi DNA Assembly Cloning Kit. Following quenching with EDTA, reactions were purified and concentrated with successive binding to SPRI beads (0.5× and 1.0×). Assembled reporter plasmids were eluted in nuclease-free water and pooled prior to transformation into freshly prepared ElectroMAX DH10B Cells (Thermo Fisher Scientific). Each transformation contained 2 µg of assembled DNA and total of 72 transformation were performed. Following recovery for 1 h in SOC medium while shaking (225 rpm, 37 °C) transformations were pooled four to a flask in 1 L of Luria Broth and then grown for 14 h under carbenicillin selection while shaking (225 rpm, 37 °C).

Reporter input libraries were purified using the Promega Pure Yield Megaprep Kit. The quality and diversity of individual pools of plasmid libraries were assessed by sequencing on an Illumina MiSeq using 50 bp paired-end reads. Estimates of library complexity were made using the PreSeq package^11^. Equimolar amounts of each plasmid library were pooled to create the final whole-genome STARR-seq plasmid library. Ten nanograms of the plasmid library was used as template to construct 12 individual STARR-seq sequencing libraries. Plasmid templates were amplified by PCR using Illumina TruSeq primers under the following conditions: 98 °C for 30 s, followed by 10 cycles of 98 °C for 10 s, 65 °C for 30 s, 72 °C for 30 s, with a final extension at 72 °C for 5 min. Libraries were subjected to 50 cycles of paired-end sequencing on the Illumina HiSeq 4000 platform.

### Assaying the whole-genome STARR-seq reporter library

A549 cells (first obtained at passage 87) were expanded under standard culture conditions using Ham’s F-12K (Kaighn’s) medium, 10% (v/v) fetal bovine serum, and 1% (v/v) penicillin–streptomycin for a total of seven passages. The whole-genome STARR-seq library was transfected into twenty-five 500 cm^2^ plates of A549 cells at ~75% confluence using Lipofectamine 3000 (Thermo Fisher Scientific) scaling the manufacturer’s recommended protocol. Each plate served as a single biological replicate. The media were changed after 24 and 48 h. Following the final media change, cells were treated with 100 nM dex (Sigma) or an equal volume (0.02% v/v) of EtOH for 0, 1, 4, 8, or 12 h. Five replicate treatments were performed for each time point. Cells were rinsed with phosphate-buffered saline (PBS) (pH 7.4) and subjected to extracellular DNase I digestion (Sigma), as described previously^7^, prior to dissociation with Trypsin-EDTA 0.25% (v/v) (Life Technologies), neutralization, and centrifugation. Cell pellets were washed with PBS and lysed in 2 mL of RLT buffer (Qiagen) supplemented with 2-mercaptoethanol (Sigma). Lysates were passed through a 18-gauge needle five times and stored at −80 °C.

### Reporter output library construction

Total RNA was recovered from cell lysates with the Qiagen RNeasy Midi Kit including the on-column DNase I digestion step. Eluates were treated with 1 μL RNase Block (Aligent) prior to poly-A RNA isolation with Dynabead Oligo-dT25 beads (Thermo Fisher Scientific) according to the manufacturet’s recommended protocol. Eight individual captures from 75 μg of total RNA were performed for each sample with subsequent processing steps carried out separately. Poly-A RNAs were treated with Turbo DNase (2 U; Thermo Fisher Scientific) and 1 μL RNase Block at 37 °C for 30 min before halting the reaction with the DNase inactivation reagent. RNA was reverse transcribed for 2.5 h at 55 °C with the STARR-seq gene-specific primer^12^ using the SuperScript III system (800 U; Thermo Fisher Scientific). Following enzyme inactivation, cDNAs were treated with RNase A (Sigma) at 37 °C for 1 h, purified with SPRI beads (2.0×), and subjected to PCR enrichment with reporter-specific primer as perviously described^12^ under the following conditions: 98 °C for 30 s, followed by 6 cycles of 98 °C for 10 s, 65 °C for 30 s, 72 °C for 30 s, with a final extension at 72 °C for 5 min. PCR products were purified with SPRI beads (1.5×) and amplified again by PCR using a standard Illumina TruSeq multiplexing oligos under the following conditions: 98 °C for 30 s, followed by 10 cycles of 98 °C for 10 s, 65 °C for 30 s, 72 °C for 30 s, with a final extension at 72 °C for 5 min. Libraries from matched samples were pooled and assessed by sequencing on an Illumina MiSeq, as above, using 50 bp paired-end reads prior to deep sequencing on the Illumina HiSeq 4000.

### Alignments and region calling

STARR-seq input library and output libraries were individually aligned to the human genome assembly hg38 with Bowtie2 (version 2.2.4)^31^, using the following parameters: bowtie2 -X 2000 sensitive. Only properly paired alignments with a MAPQ (mapping quality) score ≥30 outside hg38 centromeres, gap, and blacklist regions were retained in downstream analyses. Regions were called individually for each sample using merged STARR-seq input alignments as controls with the MACS2 package^32^ using the following parameters: -f BAMPE -g hs–ratio–keep-dup all -q 0.10. The custom scaling ratio (–ratio) provided to MACS was generated for each sample using the NCIS algorithm^33^. We generated a union set of called regions after merging any overlapping regions with bedtools (v.2.25.0)^34^. We tested for differential STARR-seq activity across the union region set by fitting negative binomial models and performing quasi-likelihood *F* -tests with edgeR (version 3.8.6)^35^. RPKM normalized STARR-seq read density was computed at single base pair resolution using deepTools utility bamCoverage^36^.

### Comparisons to other genomic datasets

We extensively compared our whole-genome reporter assay results to Hi-C, RNA-seq, DNase-seq, ATAC-seq, and ChIP-seq datasets from a time-course study of the same dex response in A549 cells^21,22,37^. Differentially expressed genes and topological associated domains were previously identified^21,22^. Analysis of ChIP-seq, ATAC-seq, and DNase-seq signals were performed using the bwtools package^38^. ChIP-seq read density (RPKM) values represent input control RPKM subtracted values, truncating the difference as zero. Post-dex averages of genomic signals represent the mean across the full dex time course as reported in ref. ^21^ (0.5, 1, 2, 3, 4, 5, 6, 7, 8, 10, and 12 h of dex treatment). ChIP-seq peak set intersections were visualized using the UpSetR package^39^.

### Motif enrichment analysis

We tested the set of 8-h DREs for enrichment of RSAT-clustered^40^ JASPAR motifs^41,42^. To do so, we first binned the dynamic regulatory elements by the magnitude of their response and controlled for baseline activity. Relative motif enrichment analysis was performed with the AME tool^43^ from the MEME suite^44^ using randomly generated dinucleotide shuffled sequences as a comparator. Motif enrichment was similarly performed on steady-state regulatory elements after separating elements into quintiles according to the reporter activity. Motif analysis of the rs10505411 alleles was performed with the MAST tool^45^ using a first-order Markov background model.

### Allele-specific dex-responsive regulatory activity analysis

To identify allele-specific dex-responsive regulatory activity, we used the mpileup tool in the samtools package^46^ to identify alignments overlapping heterozygous SNPs in the genome of GM12878 cells^14^. We further parsed variants requiring that they overlapped a regulatory region identified in this study and were covered by at least five fragments in at least five samples. In total, we tested 10,669 heterozygous alleles for differential regulatory activity in response to dex using a negative binomial model model implemented with the edgeR^35^ that included main effect and interaction terms for treatment and time. The DSS package was used to estimate dispersion^47^.

### Allele-specific ChIP analysis

We used bowtie^48^ to perform allele-specific analysis of GR, EP300, and cJUN ChIP-seq reads. Reads were aligned (bowtie -v 0 -m 1) to either the reference or alternate alleles of rs10505411, requiring no mismatches or multiple matches^16^.

## Electronic supplementary material

Supplementary Information

Description of Additional Supplementary Files

Supplementary Data 1

Reporting Summary

## Data Availability

Whole-genome STARR-seq sequencing data are publicly available through the National Center for Biotechnology Information Gene Expression Omnibus (Series GSE114063). Published ChIP-seq, RNA-seq, ATAC-seq, and DNase-seq^21^ as well as Hi-C^22^ datasets are available through the NCBI BioProject (PRJNA356880) and the ENCODE DCC portal (http://www.encodeproject.org).
