# Supplementary material for: Human genome-wide measurement of drug-responsive regulatory activity
Source: Nat Commun. 2018 Dec 21;9:5317. doi: 10.1038/s41467-018-07607-x (PMC6303339; doi:10.1038/s41467-018-07607-x)
Supplement: Supplementary file 1 — Supplementary Information [file 41467_2018_7607_MOESM1_ESM.pdf]

Supplemental Figures

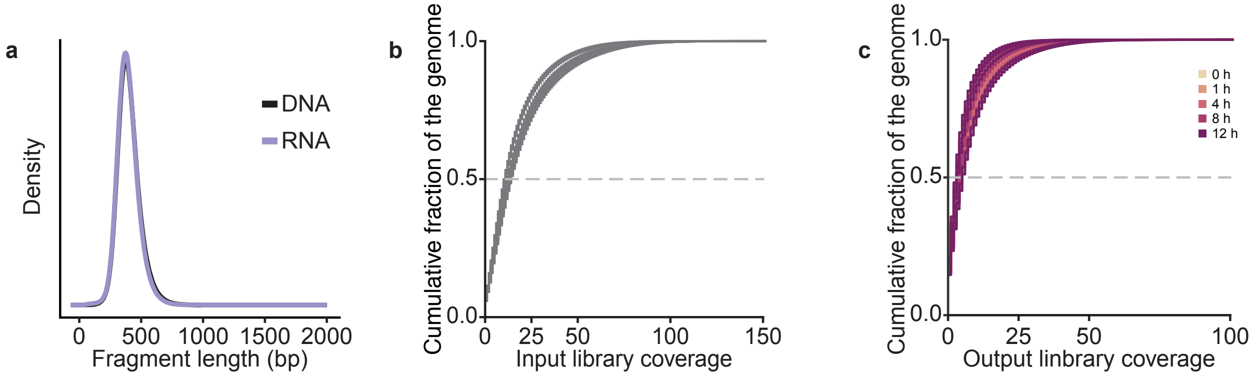

Supplementary Figure 1. Genome-wide high-throughput reporter library coverage. (a) Distribution of fragment sizes as determined by paired-end sequencing in the genome-wide high-throughput reporter input and output libraries (black and purple, respectively). (b,c) Cumulative distribution of genome-wide coverage of the individual whole genome STARR-seq input (b n = 12) and output (c; n = 25) libraries.

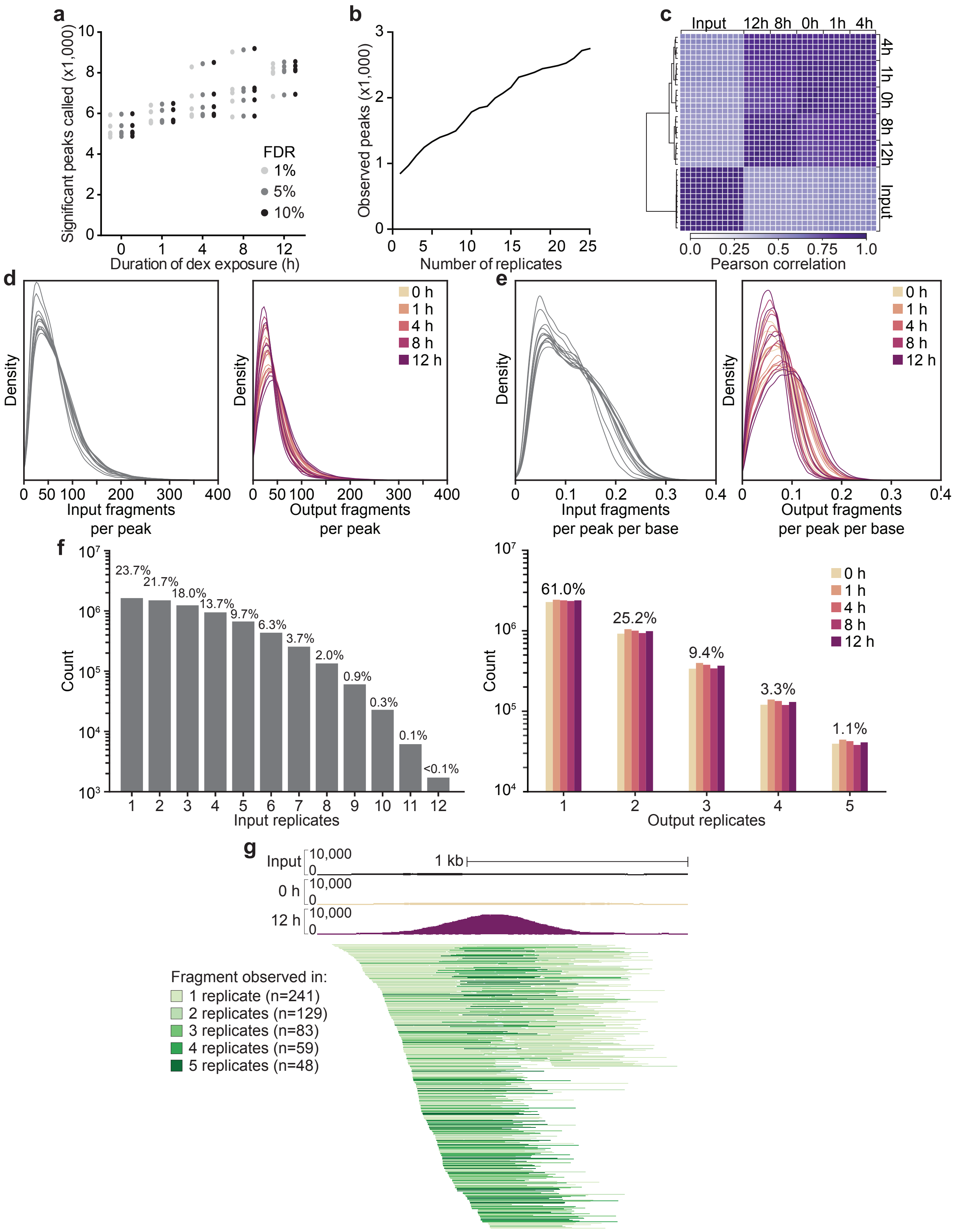

Supplementary Figure 2. Genome-wide high-throughput reporter assay metrics and

reproducibility. (a) The number regulatory elements called for all replicate output libraries with increasingly stringent FDR thresholds. (b) The union set of regions called across all replicates increased linearly with the number of replicate region sets included. Replicate region sets were randomly merged prior to counting. (c) Correlation of STARR-seq coverage for the union set of identified regulatory regions (n = 27,498). Coverage of called regulatory regions was strongly correlated within each of the five replicates for each time point and distinct from the coverage observed in the twelve input libraries. While reporter expression in these regions was highly correlated across all twenty-five output libraries hierarchical clustering of Pearson correlation coefficient values demonstrated a separation between earlier and latter time points (0, 1, and 4 hours versus 8 and 12 hours of dex exposure). (d) The distribution of fragments mapping to active STARR-seq regions in each input (left) and output (right) library. (e) As in d, normalized by the length of each STARR-seq region. The median library coverage of STARR-seq regions was 289x. (f) The frequency of input (left) and output (right) fragments mapping to STARR-seq regions are observed in replicate samples. The percentage of fragments in regions observed across replicates is provided for input libraries and the median percentage for the output libraries. (g) Fragment replication in an induced dex-responsive element. Merged coverage of the STARR-seq input, 0 and 12 hour output libraries for the upstream induced DRE identified in the IP6K3 locus (Figure 2e) are displayed in the top tracks. Individual fragments observed in the 12 hour STARR-seq output libraries are displayed below and colored according to the number of replicates that they are observed in.

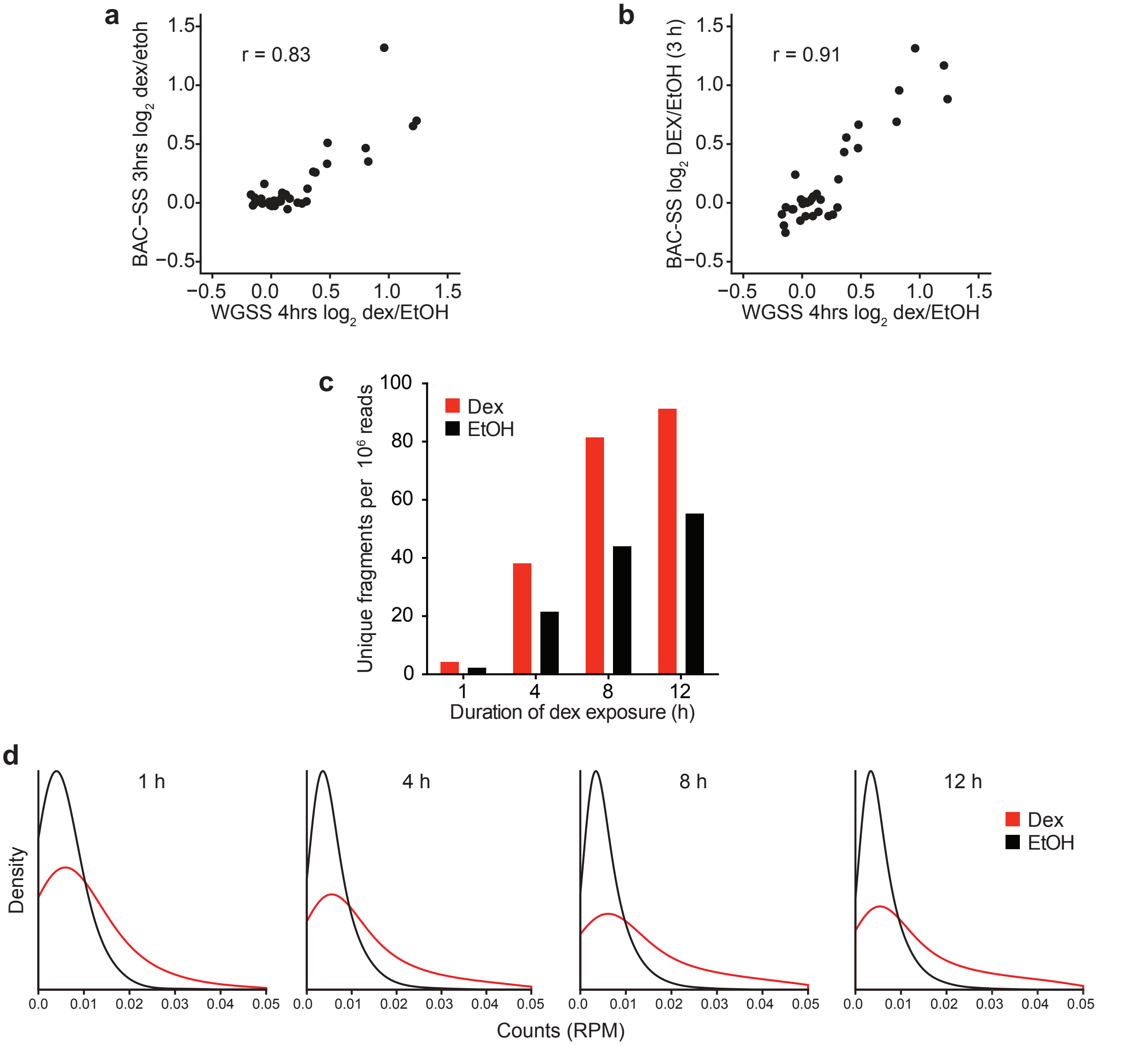

Supplementary Figure 3. Human genome scaled STARR-seq libraries recapitulate localized dex responses. (a and b) The fold change in reporter activity after three and four hours of dex treatment from BAC and whole genome STARR-seq libraries, respectively, is well correlated. BAC libraries were prepared independently[7]. (c) The number of unique fragments per million reads mapping to the top 5% of induced DREs at each time point. (c) The distribution of normalized coverage (RPM) for individual fragments mapping to the top 5% of induced DREs at each time point.

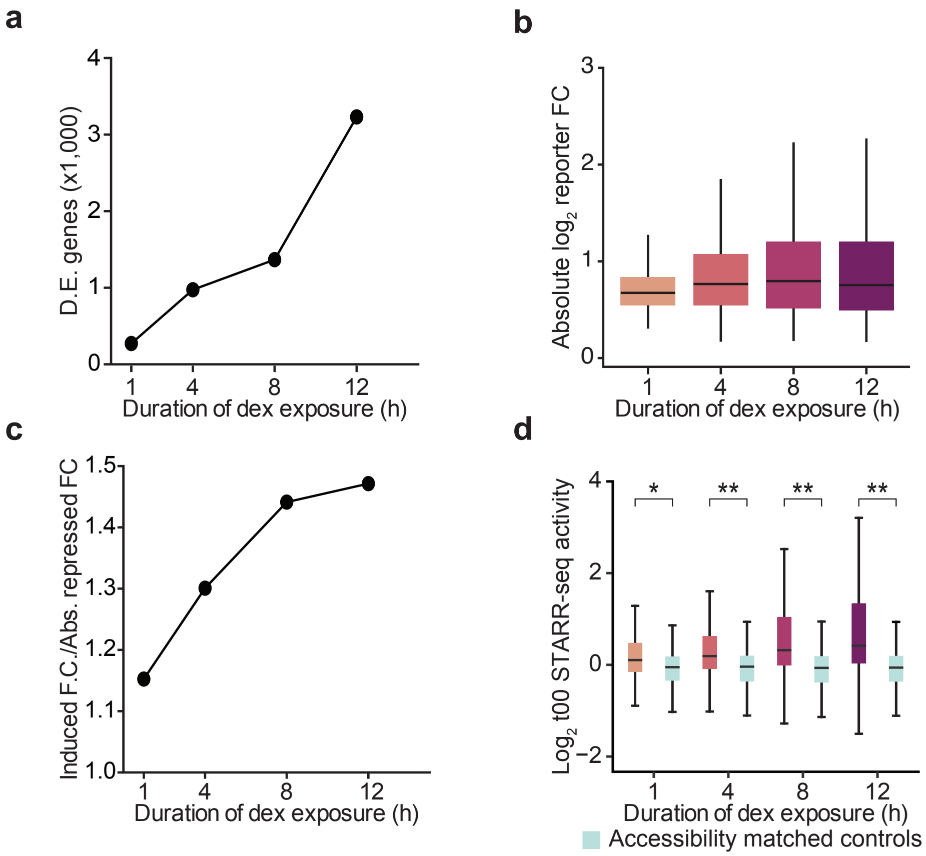

Supplementary Figure 4. Reporter activity levels. (a) The number differentially expressed genes called after treating with dex as previously reported[21]. (b) The absolute fold changes in reporter activity at dynamic DREs. (c) Induced DREs were more responsive than repressed DREs. (d) Steady state regulatory activity was enriched at induced DREs prior to dex treatment. Steady state reporter activity at induced DREs was compared to that from a set of DNase-seq accessibility matched control regions (pale blue). Mann-Whitney, * $P < 10^{-10}$, ** $P < 10^{-100}$.

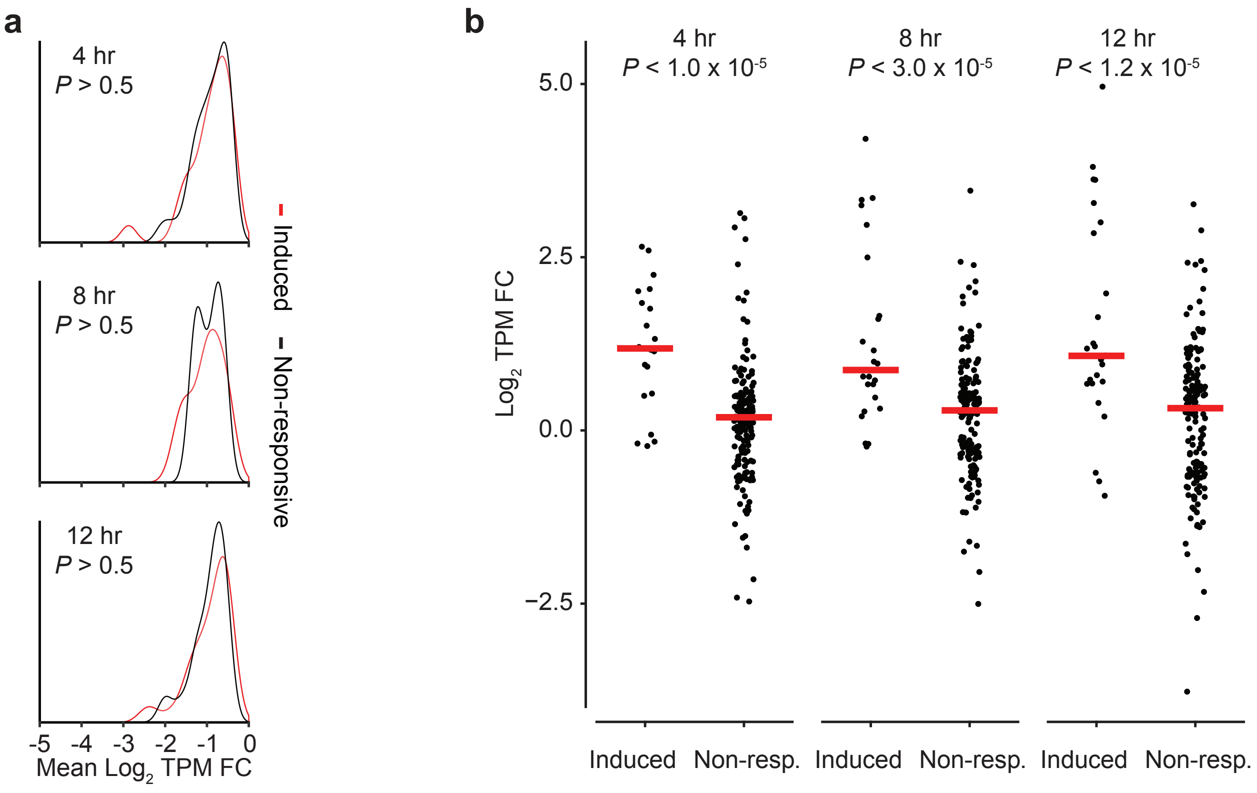

Supplementary Figure 5. Induced DREs and changes in gene expression. (a) No differences in GC-responsive gene repression were observed in TADs containing induced DREs compared to TADs containing non-responsive regulatory regions. The distribution of mean fold changes for all differentially repressed genes in the same TAD is plotted for all TADs containing an induced or non-responsive regulatory element in DHS. Median fold changes were compared with the Mann-Whitney *U*-test. (b) Differentially expressed genes with an induced DRE in their promoter exhibited greater changes in gene expression than genes with non-responsive regulatory elements in their promoters.

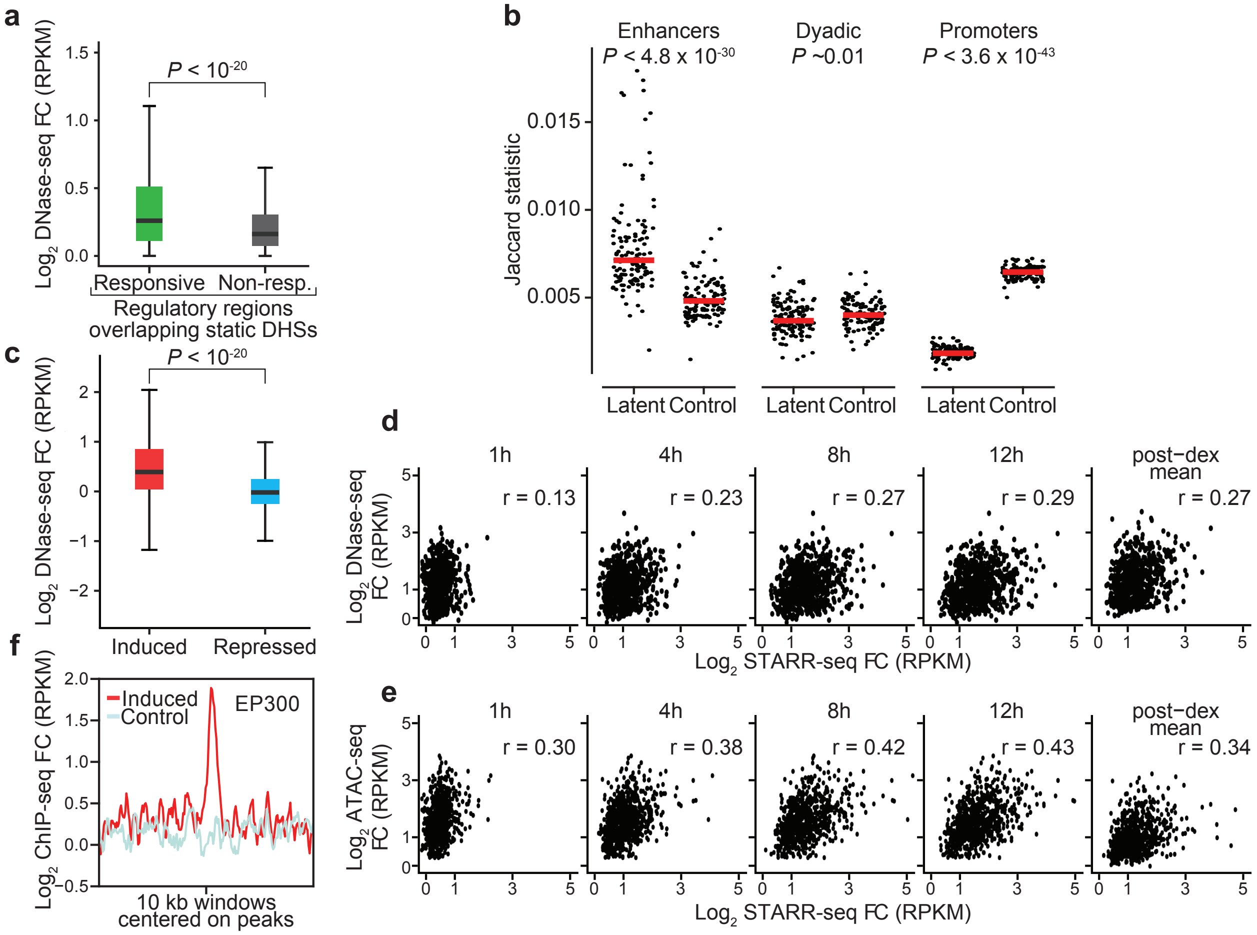

Supplementary Figure 6. Comparing genome-wide high-throughput reporter and accessibility assays. (a) DREs overlapping static DHSs had a greater dex-dependent change in accessibility than non-responsive regulatory elements in DHS. The average post-dex fold change in DNase-seq signal is plotted for each set of elements. (b) Latent regulatory elements identified by STARR-seq are enriched in putative enhancers from other cell types. The Jaccard similarity coefficient is plotted for each intersection of the set of all latent regulatory elements identified in this study and a set of putative functional elements (enhancers, dyadic, or promoters) predicted for 127 other cellular contexts by the Roadmap Epigenomics Project (http://www.broadinstitute.org/~meuleman/reg2map/HoneyBadger2_release/). Latent regulatory elements are defined as STARR-seq regions that do not overlap open chromatin in our model system. The Jaccard static was similarly calculated for a set of control regions with the same median lengths and matched dinucleotide-compositions, relative to the latent elements. Median

coefficient values are depicted as red lines. For each class of putative functional elements mean coefficient values for latent and control regions were compared with the Mann-Whitney *U*-test. Latent elements detected by STARR-seq are depleted from predicted promoters of other cell types. This reflects the remote activation of reporter expression by fragments embedded within the 3' UTR of the reporter gene. (c) Induced DREs had a greater dex-responsive change in DNase-seq accessibility than repressed DREs. (d and e) The fold change in chromatin accessibility in response to dex is plotted against the fold change in reporter activity in response to dex from averaged replicate DNase-seq or ATAC-seq and whole genome STARR-seq assays, respectively. (f) A subset of induced DREs that are not in a DHS are enriched for EP300 binding. The mean dex-treated fold change in EP300 ChIP-seq signal is plotted in 50 bp bins across 10 kb windows centered on the subset of induced DREs that do not overlap a DHS but are bound by a TF during dex treatment (n = 298) and a set of DNase-seq accessibility matched control regions (pale blue).

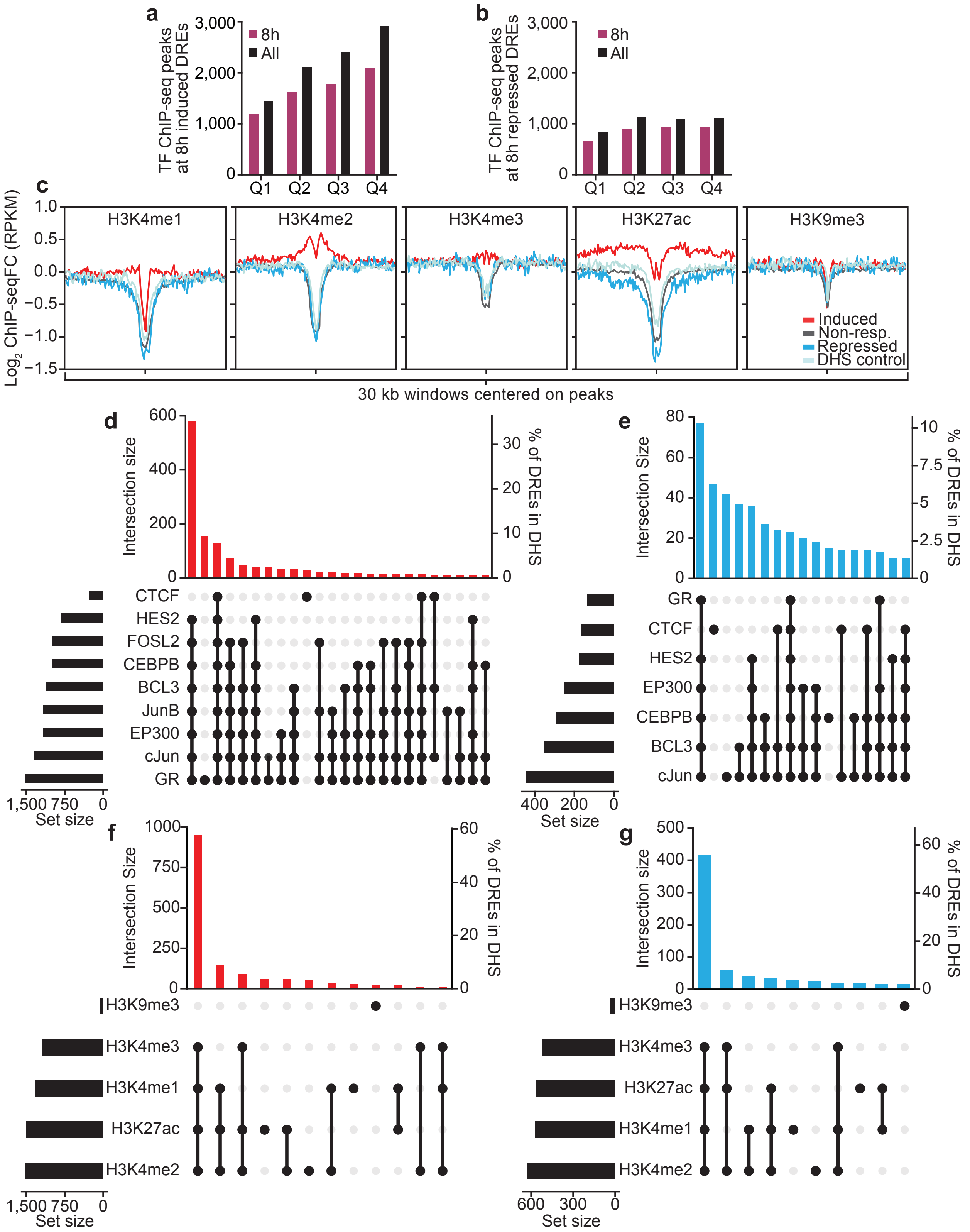

Supplementary Figure 7. Induced and repressed DREs exhibited contrasting patterns of TF binding and modified histone enrichment. (a and b) Barplots showing the number of TF binding

events identified by ChIP-seq that overlap induced and repressed DREs after eight hours of de treatment. Counts corresponding to ChIP-seq peaks observed after eight hours of treatment or at any point in the timecourse are displayed in purple and black, respectively. (c) Aggregate profile plots of the mean fold changes in post-dex ChIP-seq signal in 150 bp bins across 30 kb windows centered on DHS+ regulatory element midpoints. Induced (n = 1,646) and repressed (n = 746) DREs are shown in red and blue, respectively. Non-responsive regulatory regions (n = 6,531) are displayed in grey and control regions (n = 1,646) matched to DHS+ induced DRE accessibility are shown in pale blue. (d and e) Induced DREs are associated with increased TF binding and co-occupancy. The counts for all possible intersections of induced (d and f, red) or repressed (e and g, blue) DREs in open chromatin with nine TF ChIP-seq peak sets are displayed in bar plots and tabulated below. Sets contributing to an intersection are indicated by black dots. Intersections are sorted by number of occurrences (left y-axis). The percentage of DREs overlapping a DHS contributing to each intersection is displayed on the right y-axis. Only intersections occurring at least ten times are displayed. (f and g) Intersections with modified histone ChIP-seq peak sets, as in (d and e).

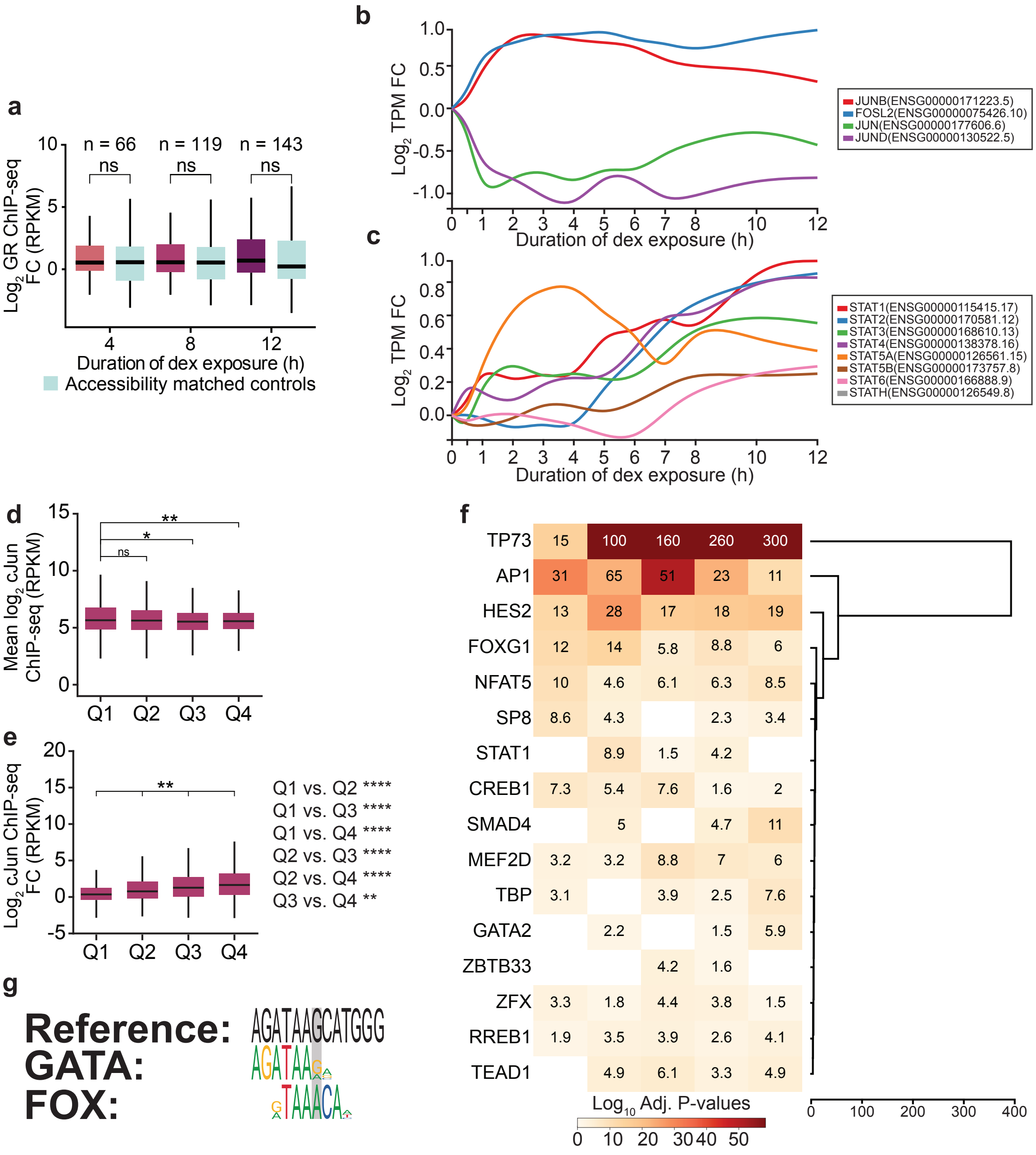

Supplementary Figure 8. Non-GR mediated dex-induced regulatory activity and motif analysis. (a) The average post-dex fold change in GR ChIP-seq signal was not significantly different at DHS+ induced DREs that lacked GR motifs and GR ChIP-seq peak calls and compared to a set of DNase-seq accessibility matched control regions (pale blue). (b,c) Changes is in gene expression for members of the AP-1 (b) and STAT (c) transcription factor families in response to dexamethasone treatment as reported in reference #21. Fold changes are relative to baseline. (d) The average steady-state cJun ChIP-seq signal is shown for induced DREs binned

according to the strength of their dex-response. Induced DREs called after eight hour of dex treatment. Steady-state AP-1 binding is greatest in the quartile exhibiting the weakest dex induction (Kruskal-Wallis test). (e) AP-1 binding is greatest at the most responsive induced DREs. The mean fold change in post-dex cJun ChIP-seq signal are plotted for the quartiles in (d). All pair-wise comparisons are significant (Kruskal-Wallis test; P-values are provided). (g) Conversion of a GATA motif to a FOXG1 motif in a drug-responsive allele-specific regulatory element. The reference allele at this position encodes a GATA3 binding motif ($P < 8.37 \times 10^{-5}$). The SNP (shaded) present in the alternative allele creates a FOXG1 motif ($P < 1.45 \times 10^{-4}$). (f) Steady-state regulatory regions are enriched for binding motifs corresponding to cell stress factors. Enriched RSAT-clustered JASPAR TF motifs[41] were identified for each quintile and then hierarchically clustered. Bonferroni corrected $\log_{10}$ *P*-values are displayed for all significant enrichments. * $P < 0.05$, ** $P < 0.01$.

Supplementary Tables

Supplementary Table 1a. Summary statistics of sequencing and alignment results for input libraries.

Sample	Sequenced Fragments	Aligned Fragments	% Aligned	Unique Alignments	% Unique Alignments	Median Unique Genomic Coverage
Input Library #1	2.35E+08	1.91E+08	81.39%	1.27E+08	66.72%	13
Input Library #2	2.43E+08	1.98E+08	81.59%	1.36E+08	68.32%	14
Input Library #3	2.56E+08	2.09E+08	81.57%	1.34E+08	64.17%	13
Input Library #4	2.25E+08	1.83E+08	81.59%	1.27E+08	69.14%	13
Input Library #5	2.35E+08	1.92E+08	81.45%	1.33E+08	69.19%	13
Input Library #6	2.18E+08	1.77E+08	81.18%	1.23E+08	69.26%	12
Input Library #7	1.98E+08	1.62E+08	81.49%	1.09E+08	67.67%	11
Input Library #8	2.23E+08	1.82E+08	81.53%	1.22E+08	66.90%	12
Input Library #9	2.46E+08	2.00E+08	81.52%	1.34E+08	67.16%	14
Input Library #10	2.46E+08	2.01E+08	81.66%	1.39E+08	69.49%	14
Input Library #11	1.69E+08	1.38E+08	81.62%	1.05E+08	76.08%	11
Input Library #12	2.19E+08	1.79E+08	81.66%	1.27E+08	70.92%	13
Merged	2.71E+09	2.21E+09	81.52%	4.86E+08	22.00%	59

Supplementary Table 1b. Summary statistics of sequencing and alignment results for output libraries.

Time point	Replicate	Cells Harvested	Sequenced Fragments	Aligned Fragments	% Aligned	Estimated Unique Fragments	Unique Alignments	% Unique Alignments	Median Unique Genomic Coverage
0 hour	#1	2.83E+07	1.22E+08	9.75E+07	79.92%	1.31E+08	5.63E+07	57.74%	5
	#2	2.75E+07	1.04E+08	8.34E+07	80.19%	9.37E+07	4.10E+07	49.13%	4
	#3	2.55E+07	8.84E+07	7.05E+07	79.70%	1.27E+08	4.30E+07	61.08%	4
	#4	4.18E+07	1.01E+08	8.03E+07	79.75%	8.35E+07	3.76E+07	46.86%	3
	#5	2.59E+07	1.08E+08	8.66E+07	80.14%	1.14E+08	4.69E+07	54.18%	4
1 hour	#1	3.29E+07	1.19E+08	9.56E+07	80.12%	1.20E+08	5.26E+07	55.06%	5
	#2	3.34E+07	1.23E+08	9.85E+07	79.92%	1.31E+08	5.40E+07	54.87%	5
	#3	3.53E+07	1.26E+08	1.01E+08	79.77%	1.23E+08	5.57E+07	55.37%	5
	#4	3.44E+07	9.41E+07	7.50E+07	79.66%	1.41E+08	4.50E+07	60.06%	4
	#5	3.49E+07	9.34E+07	7.45E+07	79.78%	1.22E+08	4.51E+07	60.53%	4
4 hour	#1	1.44E+07	1.07E+08	8.60E+07	80.14%	1.19E+08	4.73E+07	54.96%	4
	#2	2.44E+07	1.16E+08	9.26E+07	80.10%	1.48E+08	5.63E+07	60.80%	5
	#3	2.62E+07	1.00E+08	8.00E+07	79.67%	1.55E+08	4.95E+07	61.92%	4
	#4	2.42E+07	1.02E+08	8.18E+07	79.95%	8.98E+07	3.78E+07	46.17%	3
	#5	3.01E+07	1.11E+08	8.90E+07	80.02%	9.98E+07	4.13E+07	46.42%	4
8 hour	#1	3.13E+07	1.23E+08	9.85E+07	79.86%	1.23E+08	5.50E+07	55.85%	5
	#2	2.05E+07	8.06E+07	6.47E+07	80.19%	9.49E+07	3.74E+07	57.76%	3
	#3	3.29E+07	9.51E+07	7.60E+07	79.90%	1.07E+08	4.47E+07	58.85%	4
	#4	3.65E+07	9.90E+07	7.91E+07	79.90%	8.65E+07	3.39E+07	42.89%	3
	#5	3.61E+07	1.03E+08	8.18E+07	79.57%	1.19E+08	4.10E+07	50.15%	3
12 hour	#1	2.48E+07	1.46E+08	1.17E+08	79.94%	1.27E+08	6.10E+07	52.32%	5
	#2	3.95E+07	8.93E+07	7.12E+07	79.76%	8.53E+07	3.16E+07	44.36%	3
	#3	3.58E+07	9.58E+07	7.64E+07	79.71%	1.28E+08	4.04E+07	52.88%	3
	#4	3.29E+07	1.08E+08	8.64E+07	79.97%	9.26E+07	3.56E+07	41.14%	3
	#5	3.59E+07	1.34E+08	1.07E+08	79.78%	1.13E+08	5.67E+07	53.02%	5
