# Supplementary material for: Human genome-wide measurement of drug-responsive regulatory activity
Source: Nat Commun. 2018 Dec 21;9:5317. doi: 10.1038/s41467-018-07607-x (PMC6303339; doi:10.1038/s41467-018-07607-x)
Supplement: Supplementary file 2 — Description of Additional Supplementary Files [file 41467_2018_7607_MOESM2_ESM.docx]

**Title:** Supplementary Data 1

**Description:** Differential enrichment of STARR-seq regions. Coordinates, fold

change, and confidence values are provided for differential STARR-seq regions at all time points

relative to 0 hours of dex exposure.
